# Promoting regional coherence and cohesion amidst multiple assistive technology initiatives in Africa

**DOI:** 10.4102/ajod.v11i0.937

**Published:** 2022-02-10

**Authors:** Surona J. Visagie, Malcolm MacLachlan, Elsje Scheffler, Nikola Seymour

**Affiliations:** 1 Centre for Disability and Rehabilitation Studies, Faculty of Medicine and Health Sciences, Stellenbosch University, Cape Town, South Africa; 2 Department of Psychology and Assisting Living and Learning (ALL) Institute, Maynooth University, Maynooth, Ireland; 3 Olomouc University Social Health Institute, Palacký University, Olomouc, Czech Republic

**Keywords:** assistive technology, assistive products, Africa, coherence, cohesion

## Abstract

**Background:**

Appropriate provision of assistive technology services (ATS) and products are a global health issue and essential for achieving the Sustainable Development Goals (SDGs). The Sixth African Network for Evidence-to-Action on Disability (AfriNEAD) conference included a workshop on collaboration, cohesion and coherence in ATS delivery in Africa.

**Objective:**

This article aimed to summarise the workshop proceedings and to provide some recommendations on how coherence and cohesion can be facilitated in assistive technology services in Africa.

**Method:**

A round table and small group discussions on assistive technology were facilitated in the virtual space of the AfriNEAD conference. Organisations and role players in ATS and products in Africa participated as keynote speakers, round table members and in small group discussions.

**Results:**

There was consensus amongst participants that cohesive collaboration must be facilitated. They further agreed that users must be central to future action. There are local, national and regional initiatives, but none of these have grown into an African assistive technology platform. World Health Organization (WHO) Africa can bring partners together and facilitate creation, officialisation and operationalising of a continental assistive technology platform, through building on the existing initiatives. The AfriNEAD disability research country working groups can act as in-country coordinating bodies for ATS and afford a possibility of a structured approach to assistive technology research.

**Conclusion:**

It is time to break away from Western institutionalised biomedical ways of providing ATS in Africa. Africans must develop coherent, cohesive ATS driven by empowered users who build on Africa’s strengths and addresses the continents’ unique needs.

## Introduction

The provision of appropriate assistive products (AP) and assistive technology services (ATS) are essential to achieve the Sustainable Development Goals (Tebbutt et al. 2016). With their ability to enhance function, participation and quality of life, AP are enablers in multiple life areas including health, education, justice, work, recreation, culture and sports (Layton et al. 2020). As such, access to AP is a global health issue that impacts structural and intermediate social determinants of health (WHO 2010). Therefore, a workshop and round table discussion during the African Network for Evidence-to-Action on disability (AfriNEAD) conference of 2021 aimed to explore issues of coherence and cohesion of ATS in Africa. The proceedings of that workshop are presented in this article.

Globally, interest in ATS and products has grown exponentially in the last 15 years. The increasing interest in ATS started with the United Nations Convention on the Rights of Persons with Disability (UNCRPD) in 2006 (UN 2006) and the World Report on Disability in 2011 (WHO 2011). Whilst the scope of these documents was broad and not ATS-specific, important foundations were laid. The UNCRPD articles on general obligations and personal mobility, emphasise appropriateness, development, quality and affordability of AP, as well as access to information on ATS and ATS-specific training of service providers. This provided the foundation for the WHO Global Cooperation on Assistive Technology (GATE) (WHO 2015) and fruits from that alliance, including:

- The Global Priority Research Agenda for Improving Access to High-quality Affordable Assistive Technology (WHO 2017a);
- The priority assistive products list (APL) (WHO 2017b);
- Training in priority assistive products (WHO 2018, 2019);
- The Global Research, Innovation and Education in Assistive Technology (GREAT) summit meetings in 2017, 2019 and 2021;
- Assistive product specifications (WHO 2021a), and
- The forthcoming Global Report on ATS due in 2022.

There have also been important regional and international ATS initiatives through organisations such as: Rehabilitation Engineering and Assistive Technology Society of North America (RESNA), Australian Rehabilitation and Assistive Technology Association (ARATA) and the Global Alliance of ATS Organisations (GAATO). In addition, there are policy and guiding documents focusing on specific types of ATS such as prosthetics or wheelchairs or areas of service delivery such as rehabilitation (Layton et al. 2020). Resources to support supply chains (WHO 2021b) and access to appropriate ATS were also developed. Publications in scientific journals providing guidance on various aspects of ATS abound, one example being the special issue of Disability and Rehabilitation: *Assistive Technology*, volume 13(5), 2018, with a range of articles from the first GREAT summit.

Despite these high-level, up-stream initiatives, very little has changed in the lives of Africans using AP and providing ATS. The realities of little data, few policies and even less policy implementation, AP shortages, poor access, fragmented services and inappropriate provision of products remain everyday challenges on the continent. Unstable and inadequate funding, weak domestic investment, supply chain limitations and disruption, inappropriate AP and fragile logistical capacities, compounded by low prevalence of adequately trained service providers hamper ATS delivery in Africa (CHAI 2020; Edusei & Mji 2019; Matter et al. 2017; Van Niekerk, Dada & Tönsing 2019; Visagie et al. 2020).

Service providers trained in Africa and those from foreign countries are usually trained in western models of service delivery. They are well qualified to provide curative and therapeutic intervention on a one-on-one basis. But they are often not prepared for the African realities of differing cultural beliefs and practices, communities that have been repressed and silenced for ages and the sheer size, geographical, and infrastructure challenges of our continent. Poor understanding might lead to seemingly helpful interventions that have serious negative consequences and increase disease and disability over time (Mji 2019).

Through teaching, research and practice, international aid can wield a dominating influence, privileging Western biomedical models over African, community or rights-based approaches. Such dominance is ‘capacity stripping’, unjust and undermines local identities and engagement with services (MacLachlan, Carr & McAuliffe 2010), thus hindering the development of African solutions to healthcare and in this instance specifically ATS.

In the Western biomedical paradigm, the voices of users are often absent. In many instances, service users are no more than AP recipients without choice. Their opinions are neither asked nor heeded when given unsolicited and gratefulness is expected from them for the services provided by charitable donations from a paternalistic position with providers basking in their good deeds (Visagie et al. 2015).

There are examples of innovative and successful ATS delivery strategies in Africa, but little is known about them (De Witte et al. 2019). De Witte et al. (2019) found evidence of 24 (5 from Africa) ATS delivery strategies in a scoping review of low- and middle-income countries. These are often limited to specific impairments or technologies, specific regions, countries or even areas in a country. There knowledge is seldom shared, thus they cannot be scaled to other parts of the continent. Evidence on the impact and the quality of the programmes are also scarce (De Witte et al. 2019). The truth remains that most Africans do not have access to the AP that they need.

## Assistive technology services and products in Africa

The overall importance of ATS might still be lost on the African continent where health services are often in disarray and struggling to provide cure to many suffering from infectious and non-infectious diseases, poverty-related conditions and trauma related to war, violence, accidents and displacement (African Union 2016). Social welfare ministries, who are often responsible for persons with disabilities, are also struggling and are providing ad hoc products and services through community organisations parallel to and disconnected from health systems. Faith-based, and community-based organisations often step into the void and show creativity in ATS solutions. However, user training and a link to the health sector are often neglected and sustainability is challenged (CHAI 2020). Fragmentation and incoherence remain a serious barrier in the ATS sector in Africa (Bostian 2020). Responding to the COVID-19 pandemic is further stretching Africa’s already overburdened healthcare and social services (Chersich et al. 2020). The provision of rehabilitation and assistive products has been put on hold in some countries during the pandemic as it is seen as non-essential services (McKinney, McKinney & Swartz 2020).

Research in a few African countries documents the need for and access to AP, as well as funding sources, user training and maintenance (CHAI 2020; Matter & Eide 2018; Visagie et al. 2017). Some health ministries have affirmed their commitment to advocate for ATS access (Burkina Faso, United Republic of Tanzania, Republic of Kenya and Senegal). Seven countries (Ethiopia, Liberia, Malawi, Nigeria, Rwanda, Sierra Leone and Uganda) completed the WHO assistive technology capacity assessment (ATA-C) survey (CHAI 2020). A number of countries are engaged with the WHO rapid assistive technology assessment (rATA) to obtain data to understand the need, unmet need and the barriers to access ATS. The WHO and partners are supporting governments of Nigeria, Liberia, Rwanda and Sierra Leone to integrate ATS within health and social systems. Tanzania is establishing a national ATS programme and building a supply chain system of quality products and mass training of health workers. The ATS 2030 consortium is focusing most of its efforts on improving access to affordable AT in Africa.

Assistive technology was one of the commissions of the Sixth AfriNEAD conference. The conference included an ATS workshop with a focus on collaboration, cohesion and coherence in ATS delivery in Africa. This article summarises some of the ideas shared, challenges put to Africa and possible future strategies regarding ATS products and service delivery in Africa. This article aims to provide some recommendations as to how coherence and cohesion can be facilitated in AT in Africa.

## Assistive technology workshop process

A round table and small group discussions were facilitated in the virtual space of the AfriNEAD conference. Organisations and role players in ATS and AP in Africa were welcomed as keynote speakers, round table members and as participants in small group discussions. During the keynote addresses Professor Malcolm MacLachlan (from the Assisting Living & Learning Institute, Maynooth University, Ireland) provided conceptual background on cohesion and coherence. Chapal Khasnabis, on behalf of WHO-Africa, presented the current situation on ATS internationally and in Africa, leaving Africa with the challenge, ‘Where are you?’.

Round table members represented the African Community of Assistive Technology (ACAT), African Federation of Rehabilitation Professionals (FATO) and Edit Microsystems. (The representative from the Southern Africa Federation of the Disabled [SAFOD] became unavailable at the last moment.) These presentations further provided information on where we are and what is and must be done in Africa. In small group discussions, audience members and panellists discussed the questions:

- What should the priorities or initial focus of collaboration be?
- What practical strategies and platforms can be implemented to promote the sharing of information and collaboration?
- How do we ensure that AT stakeholders across the continent and wider take ownership of these strategies?

## Workshop outcomes

### Priorities and initial focus: Coherence and cohesion

Cohesion means ‘the act of forming a united whole’. In practical terms for ATS in Africa it means prioritising the linking and uniting of pockets and isolated bits of good practice together to cover a larger area. The term ‘united’ is especially powerful in the African context with its culture and history of division on one side and the philosophy of ubuntu on the other:

The concept [Ubuntu] is a basis of an African Communal life which underpins an African political, business, corporate governance, justice and conflict resolution mechanism. The concept preferably approaches any human being irrespective of his or her colour, status, ideology or origin at first as a human being. The Ubuntu philosophy puts emphasis on a human being as a being that should be treated with humanity and dignity in all matters. In the African context, the absence of Ubuntu may culminate into disorderly and crime-riddled societies (Sebola 2019:2/7).

Whilst eminent African leaders argued that Africa is politically and culturally one nation despite language and cultural divides (Sebola 2019), the reality shows a divided continent of which xenophobia is an alarming symptom (Mashau 2019; Sebola 2019). Division occurs along tribal, cultural and political lines and even more so because of the legacy of colonialism and the fallacy of white supremacy (Mashau 2019). These divisions ‘render Ubuntu homeless…and are the exact opposite of what an African community stands for’ (Mashau 2019). Where ubuntu is practiced, equality, interdependence and interconnectedness follows despite differences in race, gender and abilities. However, disability, through its contradiction of the norm, often evokes cultural and religious fear that alienates people from each other; rendering ubuntu lost (Chisale 2020). Disability therefore offers a challenge to ubuntu; at the same time ubuntu offers a powerful mechanism for countering the stigma that so often ‘others’ and marginalises people with disability.

Ubuntu must be found and brought home; it must be forged anew and will depend on the ability of individuals and groups to trust, listen and learn (Ohajunwa & Mji 2021). To truly co-construct knowledge, we need to draw on lived experiences and many different perspectives. Moral high ground, colonialism, tribalism and the various forms of violence/abuse that came with these have divided and shackled Africans for centuries. They must be explicitly acknowledged and addressed in our lives and service provision. Without doing so, we prevent respectful and meaningful collaboration (Mji 2019).

Cohesion in this fractured context implies more than sticking together pieces. We do not want to produce awkward shapes or only cover a small extent of what is needed. We want to create a united whole, with sustainable parts that support each other, in a meaningful interconnected system. Such a systemic approach must be built on principles of equity, effectiveness and efficiency with the users of assistive technology at the centre. We must find ways of working to produce processes and outcomes that chime with the African context rather than import models from elsewhere.

### Practical strategies to promote cohesion and collaboration

**FIGURE 1:**
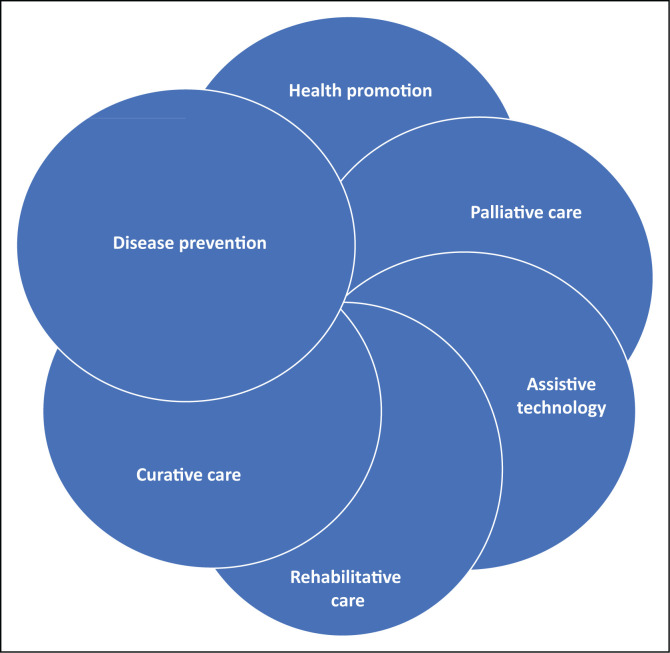
Assistive technology included amongst the strategies of primary healthcare.

At policy level the status of AT should be raised from a rehabilitation intervention (WHO 2011) to one of the key strategies of primary healthcare (PHC) as presented in Figure 1.

Recognition of the legitimacy of ‘assistive living’ as an authentic mode of being a valued and contributing member of society, is key to creating this shift from AT being a rehabilitation-related intervention to being integrated in mainstream healthcare and well-being (Khasnabis, Holloway & MacLachlan 2020; Khasnabis, Mirza & MacLachlan 2015). Human and technological assistance to perform daily activities in the case of impairment and functional decline should be given in the same manner as preventative practices such as vaccination and curative practices such as medication are a given. A similar suggestion was that AT products are classified as essential health products. Thus, their profile should be raised to be as important as that of vaccination, medication and diagnostic interventions such as x-rays. At a philosophical and organisational level, AT must be recognised as a core component of health, social care, educational and work support services.

Furthermore, for AT to meaningfully contribute to population health and wellness, population health itself must be improved (African Union 2016). Primary healthcare in Africa suffers from poor government commitment and investment, fragmentation, limited personnel, resources and infrastructure and low status (Mash et al. 2019). However, there has been a resurgence of interest in Africa to strengthen PHC through community-oriented primary care (COPC). Community-oriented primary care is ‘an approach to delivering PHC that integrates primary care practice and public health for a defined community’ (Mash et al. 2019). Community-oriented primary care can also be the vehicle for integrating AT in PHC. The nine key principles of implementing COPC, (1) a defined community, (2), a multidisciplinary team approach, (3) a comprehensive approach, (4) an equitable approach, (5) analysis of local health needs and assets, (6) prioritisation of health needs and interventions, (7) community participation, (8) evidence-based and scientific, (9) service integration around users, resonate closely with some of the key requirements to facilitate AT service provision (Mash et al. 2019).

The WHO APL was intended as a catalyst to ensure availability of essential assistive products to all (WHO 2017). The APL is supported by training programmes for healthcare and community workers (WHO 2018, 2019). However, currently, limited local production and fragile supply chain systems limit access and availability to these essential products in Africa (CHAI 2020). Available products may not be appropriate or of poor quality. Access to appropriate assistive devices may be facilitated through global initiatives such as: WHO assistive product specifications (2021a), a public procurement manual (2021b) and including AT in the UNICEF catalogue. Recently, the process to include hearing aids and wheelchairs was started. To ensure appropriateness of products, design must factor in feedback from users in Africa.

To go about service development in a coherent manner, silo of service provision, in-fighting and territorial boundaries must be recognised, named and dismantled. Assistive technology services must be provided in ways targeted at user needs, less dependent on the availability of certain health professionals and able to utilise community resources. Identifying skill sets in terms of competencies rather than professions ‘staff skills not staff types’ is one way to address the shortage of Western-styled health professions (MacLachlan, Mannan & McAuliffe 2011).

Assistive product users and the communities they live in have knowledge and resources. They must become partners in ATS delivery. Industry must facilitate co-design and production initiatives in communities. Users must be included in the AT workforce and assisted to deliver peer-led information and support programmes (Layton et al. 2021). Where appropriate, the task of assessment and prescription, fitting, training and maintenance should gradually be shifted from professionals to appropriately trained users, (Layton et al. 2021) community healthcare workers, and community-based rehabilitation workers (Visagie et al. 2020).

Appropriate devices and technology must reach and enable the majority. The WHO recommends that design specifications of technically complex products meet the needs of as many users as possible and provide options for adjustment and customisation (WHO 2021a). It is generally accepted that with mainstream products (smartphones, computer technology and software) more people can be assisted. However, availability and functionality of these information technology products are not a given in Africa. Their cost is an inhibiting factor in poor communities. Infrastructure limitations and data costs further hinder their use in Africa (Allsop, Namisango & Powell 2018; Visagie et al. 2019). Other mainstream products such as quadbikes for mobility might be something to consider, but costs might also prove inhibitive for their use.

Research on ATS in Africa must be relevant to Africa and African communities and users (Swartz 2014). Some Western research epistemologies, concepts and strategies might be unsuitable and devalue Africa, its people and its knowledge (Meekosha 2008). Research methodologies must be culturally responsible and based on social justice. Participants and researchers must collectively and collaboratively contribute to change through equal participation and inclusiveness. Participants’ worldview must be expressed and their narratives must be passed on. Research should empower them to speak and do for themselves. Previous repression, inappropriate interventions and disruption of indigenous processes and practices with severely negative outcomes for indigenous people have left them distrustful of foreigners and foreign interventions (Mji 2019). Thus, researchers entering African spaces must do so with humility, respect and a conscious focus on freeing the voices and ways of local communities. ‘Indigenous communities have critical knowledge and understandings to share that can enhance or contribute to the research process and outcomes…we had to listen and learn, more than talk’ (Ohajunwa & Mji 2021:4 & 5).

World Health Organization AT research resources such as rATA and ATA-C may be used where appropriate, but they follow quantitative positivist epistemologies where research participants are mere participants. Whilst these processes might empower a few AT users as research assistants, they will not facilitate participative practices, co-construction of knowledge and moving forward together. To be culturally and contextually relevant, generic quantitative instruments, measuring ‘how much’, must be interpreted with complementary discursive research methods – providing insights into meanings and interpretations. Participative and emancipatory approaches such as active co-design combined with the commitment to transform assistive service delivery can result in improved service outcomes and recognising the role and expertise of AT users (Layton et al. 2021).

In addition, financial barriers hamper access to academic journals and the information contained therein. Open access journals have prohibitive publication fees leaving African academics with the choice of either paying the fees or publishing in journals that is less accessible.

### Information sharing to enhance ownership

Sharing platforms already exist in Africa, but these, like service delivery strategies, are not cohesive and coherent. Some have a specific focus such as African Journal of Disability (AJOD) and Advancing Disability Research in Africa (ADIRA). Others function in a specific language, such as the African Community of Practice on Assistive Technology (ACAT). Or they operate in certain countries only (SAFOD) or are mainly focused on one sub-set of persons interested in AT (FATO). Collaboration amongst the various bodies is developing. For instance, the quarterly newsletter by ACAT is translated into French by FATO to ensure wider sharing of information. These bodies have a role to play and there might be a need for even more sharing platforms as one size will not fit all. However, these platforms should, like an umbrella with different panels, fit together into a coherent whole and promote cohesiveness. There is therefore a need for a coordinating body for AT in Africa, which should track, collate and make available information from various platforms.

Sharing and networking strategies must be relevant, appropriate, accessible and active. They must be responsive to feedback and have a representative governing body. Activity can probably be related to relevancy and appropriateness as people use what they find relevant and appropriate. However, use might be hampered by access. Platforms will function mostly in virtual spaces to save time and money, and be more inclusive of all. But as already indicated many African AT users struggle to get access to virtual spaces (Allsop et al. 2018; Visagie et al. 2019).

The AfriNEAD, a regional disability research network, provides a platform for networking amongst disability researchers, organisations of disabled people (ODPs), government, business and civil society. Its main goal is to use research evidence to impact on policy and practice to effect change in the lives of persons with disability. More than 20 African countries are affiliated to the network. The AfriNEAD is developing Disability Research Country Working Groups (DRCWGs) as an in-country structure to coordinate disability research. These groups might be a resource that can assist with appropriate ATS research in Africa.

## Recommendations

Specific recommendations include:

- Developing a research agenda for Africa, which should be supported by funding for the research and dissemination of findings
- Facilitating strategies to include users in development, design, manufacturing and all service steps
- Facilitating the development of a diverse suitably trained provider corps
- Identifying and upscaling of regional good practice clinical models
- Supporting existing network strategies to develop and come together under one umbrella

Workshop participants suggested the creation, officialisation and operationalisation of a continental AT platform in Africa. World Health Organization Africa can bring partners together and facilitate the identification and development of such a collaborative body through building on the existing initiatives. The AfriNEAD DRCWGs could function as in-country coordinating bodies for AT and afford a possibility for a structured approach to AT research. The AfriNEAD mother body can identify AT experts to assist in training on participative and emancipatory research skills. World Health Organization Africa can provide training and guidance on how to source funding.

## Conclusion

It is time to break down the walls of Western institutionalised biomedical ways of providing AT services in Africa. We must stitch together the fragments of AT research and service provision in Africa to create a cohesive, coherent unit through effective and meaningful collaboration. Assistive technology users must be at the centre of the process. We must revisit our AT practices and move from our comfort zones. If we continue in our old ways, we will continue to exclude most Africans who need AT. We must look outside our current structures, organisations and systems, and re-imagine an African way of providing AT, which is contextually and culturally appropriate and tailored to resource realities. Through a functional, innovative lens, the potential for people with disabilities in need of AT can be realised in Africa.
